# Professional Advertising

**Published:** 1883-11

**Authors:** 


### Editorial.

### Professional Advertising. Is it Ethical?

The profession have been guided in their relations with the public, not only by the code of medical ethics, but by the unwritten law, deeply impressed upon its individual members, and by long-established custom regarded with a reverence which amounts to a real conviction in their utility and necessity. Any attempt to parade individual skill or attainments before the public, through the secular press, has been wisely discountenanced, not only as opposed to ethical rules, but to the general sentiment of the profession. Our attention has been, of late, directed to reports of operations, flattering mention of professional skill, the introduction of new methods of surgical procedure, published in the daily press, evidently from the pen of one familiar with the use of technical terms. It is charitable to suppose that an enthusiasm born of admiration of the new candidate for professional and public honors may have inspired the first laudatory notice given by the daily press, but a repetition of the same methods, in successive issues of the same paper, impresses us with the conclusion that there is a fixed method in the course of the new aspirant, which savors somewhat of professional advertising, to which, as journalists, we feel it our duty to call attention.

Our city in its commercial as well as in its professional interests has been wisely conservative, and as a consequence we are sensitive to innovations upon old-established customs. Those indoctrinated with the habits and methods of other sections may ignorantly endeavor to use the license, tolerated elsewhere, of tickling the popular ear through the columns of the daily press, and thus establish a reputation by methods which are used by charlatans, and long since discountenanced by the better sentiment of the profession in older communities. It matters not, however, what the motive has been, which has prompted this practice, in the special case under consideration. The question is presented, is it ethical or strictly professional to make use of the secular press to publish such statements in regard to operations and to portray special methods of surgical procedure? Looking beyond the fact itself, who is intended to be benefited by such a course? What do the public know of antisepsis in surgery, of skin grafting and other modern ideas in which the merest tyros in medicines are now instructed. Is there not rather a motive to parade the name of the surgeon before the community and thus strive to establish a reputation by objectionable devices. Would it not be more professional to trust to superior ability, and attainments, and skill, which are sure to bring success to the possessor in the course of time? Rushing into print may not be the surest way of rushing into practice, and it is not always prudent to violate the ethical sensibilities of the profession with a view to gain time or to hasten the ordinary course of human events.

We are inclined to regard with disfavor these imported ideas for the acquisition of fame, or reputation, or practice, for the reason that they are not in accordance with our notions of medical ethics. We submit the question if it is in good taste for the successor of Hamilton and Moore, as well as for the institution with which he is connected, to attempt to rival the World's Dispensary in heralding his skill or sounding the praises of the panaceas offered to rescue human life or to alleviate human suffering. The medical profession offers to its votaries a more respectable pathway to fame and success.

We do not judge of this matter in a narrow or prejudiced manner. As journalists we criticise only for the good of the profession, with a view to antagonize any effort for selfish ends which will drag it down to the level of charlatanry.

Note.—I desire to express my dissent from the above editorial, so far as it may seem to imply that the lecturer on surgery in the Buffalo Medical College was privy to the publication of the articles referred to. I can not believe that a gentleman, by birth and education could be so devoid of professional instinct. At the same time, I fully agree with my confreres that our duty as journalists will not permit us to ignore so gross a violation of professional ethics. It behooves the medical authorities of the Buffalo Medical College and Hospital to prevent the repetition of such a scandal. If their discipline is too lax to effect that, they must assume the responsibility for the results of their own weakness and inefficiency. A. R. D.

